# Reversibility of membrane permeabilization upon pulsed electric field treatment in *Lactobacillus plantarum* WCFS1

**DOI:** 10.1038/s41598-019-56299-w

**Published:** 2019-12-27

**Authors:** E. M. J. Vaessen, R. A. H. Timmermans, M. H. Tempelaars, M. A. I. Schutyser, H. M. W. den Besten

**Affiliations:** 1 Food Process Engineering, Wageningen University and Research, P.O. Box 17, 6700 AA Wageningen, The Netherlands; 2 Food Microbiology, Wageningen University and Research, P.O. Box 17, 6700 AA Wageningen, The Netherlands; 3 Wageningen Food and Biobased Research, Wageningen University and Research, P.O. Box 17, 6700 AA Wageningen, The Netherlands

**Keywords:** Microbiology techniques, Applied microbiology

## Abstract

Pulsed electric field (PEF) treatment, or electroporation, can be used to load molecules into cells. The permeabilizing effect of the PEF treatment on the cellular membrane can be either reversible or irreversible depending on the severity of the PEF treatment conditions. The influence of PEF on the reversibility of membrane permeabilization in *Lactobacillus plantarum* WCFS1 by two different fluorescent staining methods was investigated in this study. Whereas staining with propidium iodide (PI) before and after PEF treatment indicated small reversible permeabilized fractions of maximum 14%, the use of a double staining method with PI and SYTOX Green suggested larger reversible permeabilized fractions up to 40% of the population. This difference shows that the choice for a fluorescent staining method affects the conclusions drawn regarding reversibility of membrane permeabilization. Additionally, the effect of PEF treatment conditions on membrane integrity was compared, indicating a relation between critical electric field strength, cell size and membrane permeabilization. Overall this study showed the possibilities and limitations of fluorescent membrane integrity staining methods for PEF studies.

## Introduction

Electroporation, or pulsed electric field (PEF) treatment, is currently widely used for many applications in several cell types, including bacteria, microalgae, mammalian cells and plant cells or tissue^1,2^. The reason can be to inactivate cells, to extract components from cells or to load cells with components such as DNA or small molecules. All these applications employ the permeabilizing effect of the electric field on the cell membrane. A PEF treatment is applied to the cells in a conductive medium between two electrodes using one or more high voltage pulses with a pulse duration in the nanosecond to millisecond range^3^. The electric field strength applied between the electrodes influences the transmembrane potential difference over the cellular membrane. When this potential difference exceeds a critical value, pore formation occurs in the membrane of the cells^4,5^. Depending on the treatment conditions, pore formation in the cell membrane can be either reversible or irreversible. Loading of cells with DNA or small molecules requires reversible electroporation and survival of the electroporated cells, whereas inactivation requires irreversible electroporation leading to cell death^6^.

Loading of small molecules, such as trehalose, into cells has been shown to increase the processing robustness of mammalian cells and plant tissue^7,8^. Our previous study demonstrated that PEF treatment can also be used to increase intracellular trehalose in the model probiotic bacterium, *Lactobacillus plantarum* WCFS1, while maintaining culture viability^9^. However, for enhanced robustness during further processing of this probiotic bacterium, the PEF treatment should be further optimized to increase the fraction of reversible electroporated cells loaded with trehalose^10^. In order to achieve this, more knowledge is required related to PEF processing parameters and their effect on reversibility of membrane permeabilization.

A common method to detect bacterial membrane permeability is staining of cells with propidium iodide (PI). PI is a small (668 Da) hydrophilic fluorescent probe that can enter the cell only when the membrane integrity is compromised, for example because of pore formation during PEF treatment^11^. When it binds to nucleic acids inside the cell, the red fluorescence of PI increases 20 to 30 fold, making it a valuable tool to detect membrane permeability and in some cases cell viability^11^. Many studies used this method to detect irreversible pore formation in bacteria by addition of the fluorescent stain after the PEF treatment^12–15^. Furthermore, some studies have used it to detect reversibility of pore formation in both Gram-positive and Gram-negative bacteria to study inactivation mechanisms of the PEF treatment^11,16^. In these studies, PI was added before and after PEF treatment to study membrane permeabilization in relation to loss of viability for inactivation purposes.

In addition to PI staining, also other fluorescent stains have been used to study the effect of electroporation on microbial cells. One of these stains is cFDA (carboxyfluorescein diacetate), which is a probe for esterase activity and is therefore used as an indication for cell viability after PEF^17^. Furthermore, respiratory activity is sometimes employed as a measure of cell viability by using fluorescent redox probes and has been studied in relation to PEF as well^18^. The results obtained with different stains cannot always be compared as they can focus on other aspects of cell viability^19,20^. Therefore, we use in this study a second stain with a similar function as PI, namely SYTOX Green. SYTOX Green is also a membrane integrity stain, which binds nucleic acids after entering a cell and has been used to study membrane permeability after PEF treatment as well^21,22^. A recent study also showed that not only the cellular membrane is affected by PEF, but also the cell wall material^23^. This cell wall itself however, consisting of peptidoglycan layers, is not expected to be a barrier for molecules with the size of PI or SYTOX Green^24^.

The aim of this study is to understand the reversible permeabilization of the cellular membrane upon PEF treatment for the uptake of molecules in *Lactobacillus plantarum* WCFS1. To study the reversibility of membrane permeabilization two different methods were used; firstly the addition of PI before and after PEF treatment and secondly, a combination staining of PI and SYTOX Green. The first method has the advantage of using only one membrane permeability probe, with the disadvantage that two parallel PEF treatments are required. For the second method this was the other way around, the advantage is that only one PEF treatment is required and more analysis options are possible on the different fractions using flow cytometry. The disadvantage of the second method is that two different markers for membrane permeability are used. These two staining methods are compared for different PEF treatments, and in addition the effect of the different PEF treatments on membrane permeability is discussed.

## Materials and Methods

### Culture preparation for electroporation experiments

*Lactobacillus plantarum* WCFS1, originally isolated from human saliva^25^, was obtained from the Food Microbiology strain collection. For every PEF experiment a fresh stationary phase culture of *Lactobacillus plantarum* WCFS1 was used. *L. plantarum* WCFS1 was cultured in the same way as described in previous research^9^. Briefly, fresh cultures were prepared from a frozen stock culture (−80 °C) on De Man Rogosa and Sharpe (MRS) (Merck, Germany) agar (Oxoid, United Kingdom) plates. Plates were incubated for 65–70 h at 30 °C after which the plates were stored at 4 °C until further use for a maximum of three days. A single colony was used to inoculate 10 mL MRS broth and incubated statically for 24 ± 2 hours at 30 °C. After incubation, the culture was diluted 1:100 into MRS broth and incubated statically for 16–18 hours at 30 °C. This stationary phase culture (9.5 log CFU/mL) was centrifuged for 10 minutes at 13,500 × *g* at 20 °C, and the resulting pellet was washed once with washing solution and subsequently suspended in electroporation medium, containing 0.3 M trehalose (Merck, Germany). The exact compositions of the washing solution and electroporation medium can be found in previous research^9^.The final conductivity and pH of the suspended culture (9.5 log CFU/mL) in electroporation medium at 21 °C were 0.15 S/m and pH 6.8, respectively.

### Electroporation equipment and settings

Electroporation was performed in disposable cuvettes with aluminium electrodes and an electrode distance of 2 mm using Gene Pulser Xcell equipment (Bio-Rad, USA), including the PC mode (Bio-Rad, USA). For all experiments, 400 µL of culture at room temperature (±21 °C) was subjected to a square wave protocol with different voltages, pulse durations and number of pulses. The following voltages were used: 500, 1000, 1500, 2000 and 2500 V resulting in electric fields of 2.5, 5.0, 7.5, 10.0 and 12.5 kV/cm. Pulse duration was set to 50, 100 or 1000 µs and the number of pulses to 1, 2, 4 or 10. The pulse interval of the equipment between two pulses was 5 s by default. Multiple pulses were triggered by manual pressing the pulse button, leading to a pulse interval of 5–10 s. Resulting droop values (average decay of pulse height) were monitored and were at maximum 6% for pulses up to 100 µs duration and approximately 18% for pulses of 1 ms duration. These droop values were used to correct the electric field strengths that were used to calculate the specific energy input of the electroporation treatment according to Eq. 1. Here, *w* represents the specific energy input (J/kg), σ the culture conductivity (S/m), *E* the electric field strength (V/m), *τ* the pulse duration (s), *n*_*p*_ the number of pulses and ρ density of the medium (kg/m^3^). For the density a value of 1039 kg/m^3^ was used, which is the density of a 10 (w/w)% sucrose solution^26^. It is assumed similar to the density of our electroporation solution with approximately 10% trehalose.

1 $$w=\frac{\sigma \cdot {E}^{2}\cdot \tau \cdot {n}_{p}}{\rho }$$

Additionally, the maximum theoretical temperature increase was calculated on the basis of the energy input according to Eq. 2, in which Δ*T* is the temperature increase (K) and *c*_*p*_ the heat capacity of the liquid (J/kg·K). A heat capacity of 3950 J/kg·K was used for the 10 (w/w)% trehalose medium, again similar to that for a 10 (w/w)% sucrose solution^26^.

2 $$\Delta T=\frac{w}{{c}_{p}}$$

### Fluorescence staining

**Figure 1:**
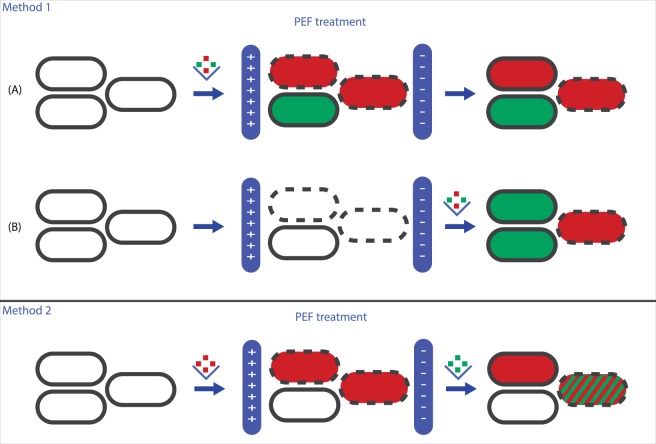
Schematic overview of the two staining methods used in this study. Dashed black lines represent damaged/permeabilized membranes and intact black lines represent intact cell membranes. In method 1 a mixture of PI (red) and counterstain SYTO 9 (green) was added either before PEF treatment (**A**) or after PEF treatment (**B**). Reversibility of membrane integrity is based on the difference in PI uptake between A and B. In method 2, the PI stain (red) was added before PEF and SYTOX Green (green) after the PEF treatment. Reversibility of membrane integrity is based on PI and/or SYTOX Green uptake. Coloured squares above the arrows indicate the moment in the procedure when the stain(s) were added to the culture.

Analysis of membrane permeability was based on cell membrane permeability for the membrane impermeable stains propidium iodide (PI) and SYTOX Green (Invitrogen, USA). Several staining methods have been used for the different experiments. In the experiments with PI addition before and after PEF treatment, a counterstain is used to visualize all cells under the fluorescent microscope. This counterstain, SYTO 9 (Invitrogen, USA), is a nucleic acid stain able to penetrate intact membranes colouring all cells green. PI has a stronger nucleic acid binding affinity and can displace SYTO9 when entering the cell, therefore once cells take up PI, the red fluorescence of PI is observed under the microscope^27^. A graphical overview of the two staining methods is shown in Fig. 1. The following sections will describe these methods in more detail.

#### Addition of PI and counterstain SYTO 9 before or after electroporation

The experiments with the addition of PI and counterstain SYTO 9 before or after electroporation were carried out with the use of a LIVE/DEAD staining kit (Invitrogen, USA). This kit contains the membrane permeable stain SYTO 9, which stains all cells green and the membrane impermeable stain propidium iodide (PI), which exhibits a bright red fluorescence signal upon binding to nucleic acids after entering the cell with permeabilized membrane. A culture suspended in electroporation medium was split into two samples, as indicated and visualised in Fig. 1-method 1. In the first sample (indicated as A) the stains were added before electroporation, and part of this sample was also kept as a control (without electroporation). In the second sample (indicated as B), the PI and SYTO 9 stains were added after electroporation. The final PI and SYTO 9 concentrations in both samples were 40 µM PI and 3.3 µM SYTO 9. Analysis of membrane permeability was performed with fluorescence microscopy after at least 10 minutes of incubation at room temperature in the same way as described previously^9^. Briefly, 10–20 images with about 100–400 cells per image were captured per sample and subsequently the red and green cells on these images were counted using an image analysis routine as described by Perdana *et al*.^28^. The percentages of green cells on the images were averaged for each sample (control, electroporated with stain, stain added after electroporation) and subsequently these averages were used to determine the fractions of the culture that were already permeable before electroporation, irreversibly permeated, reversibly permeated or not permeated as described in Table 1.

**Table 1:** Determination of the unaffected, reversible, irreversible and initially permeabilized fractions of the cell population upon PEF treatment as analysed by the two different fluorescence staining methods.

	Method 1 PI before and after PEF with counterstain SYTO 9	Method 2 PI before and SYTOX Green after PEF
Not permeabilized by PEF (unaffected, intact membrane)	% green cells in sample stained before PEF	% cells low in PI and low in SYTOX Green fluorescence
Reversible permeabilized cells	% green cells in sample stained after PEF minus % green cells in sample stained before PEF	% cells high in PI, low in SYTOX Green fluorescence
Irreversible permeabilized cells	% green cells in control sample minus % green cells in sample stained after PEF	% cells high in PI and high in SYTOX Green fluorescence
Cells already permeabilized before PEF	100% minus green cells in control sample (no PEF)*	n.a*

*The fraction of cells already permeabilized before PEF was checked for every experiment in a control sample and was <2% for all cultures.

#### Addition of PI and SYTOX Green

In the experiments with PI and SYTOX Green, both stains were added to the same electroporation cuvette at a different moment in the procedure (Fig. 1-method 2 for graphical overview). Before electroporation PI (Invitrogen, USA) was added to the culture in electroporation medium in a final concentration of 40 µM. Subsequently the sample was electroporated and kept at room temperature for approximately 10 minutes after electroporation, after which SYTOX Green (Invitrogen, USA) was added to the sample in a final concentration of 5 µM. Electroporated and control samples were subsequently analysed using a BD-FACS Aria III flow cytometer (instead of fluorescent microscopy) to be able to detect cells that took up both PI and SYTOX Green. First, the single cells were selected and determined using forward scatter (FSC) and side scatter (SSC) parameters. Second, 50,000 events per sample were analysed for their fluorescent properties using a 488-nm laser with a 502LP and 600 LP (selection range 502 to 600-nm) filter set for SYTOX Green and a 561-nm laser with a 600LP and 610/20 filter set for PI. The analysis of the different populations in the density plots was based on the PI and SYTOX Green fluorescence intensities as described in Table 1. To evaluate whether the use of flow cytometry instead of fluorescent microscopy imaging made a difference for the results obtained regarding membrane permeability, some experiments with PI addition before and after PEF treatment (method 1) were repeated and analysed with the flow cytometer and the flow cytometer gave similar results compared to the fluorescence microscopy analysis with respect to the percentages of unaffected, reversible and irreversible permeabilized cells.

### Survival analysis and selective plating

In order to compare fluorescence staining results to culturability of the PEF treated cells, additional PEF experiments were performed and followed by plate counting. PEF treated and control samples were decimally diluted in phosphate buffered saline (PBS), plated on MRS agar plates and incubated under microaerobic conditions at 30 °C for 2–4 days^9^. To determine the sublethally damaged fraction of cells that cannot grow out when an additional hurdle is present, MRS agar plates supplemented with 5 (w/v)% NaCl were also prepared. Control and PEF treated cultures were plated on both MRS and MRS-NaCl plates. These plates were incubated at 30 °C under microaerobic conditions for 3–5 days. After incubation, plates were counted and the counts of six replicate plates of each sample were averaged and used to determine the CFU/mL of the sample. Survival was calculated as a percentage of CFU/mL of the PEF treated sample compared to the control sample.

### Heat treatment

Heat treatment experiments were performed to evaluate whether a temperature of 45 °C, which is the maximum theoretical temperature during PEF experiments, would result in PI uptake. Small volumes (50 µl in a 1.5 ml Eppendorf vial) of *L. plantarum* WCFS1 cultures in trehalose medium were incubated for 1 and 5 minutes in a water bath at 45 °C in the presence of 40 µM PI and 3.3 µM SYTO 9.

### Experimental set-up

All experiments were carried out at least in biologically independent duplicates, performed on different days with another pre-culture. For each independent experiment, the measured result was again obtained by analysis of multiple cells or samples (for example, ~2,000–5,000 cells for fluorescence microscopy, 50,000 cells for flow cytometry and six replicate plates for plate counting). Average values of the independent replicates are presented with error bars indicating the standard deviation of these biologically independent replicates. Significance was tested with a Student’s *t*-test, using a P value of 0.05.

## Results and Discussion

### Assessment of reversibility of membrane permeabilization

**Figure 2:**
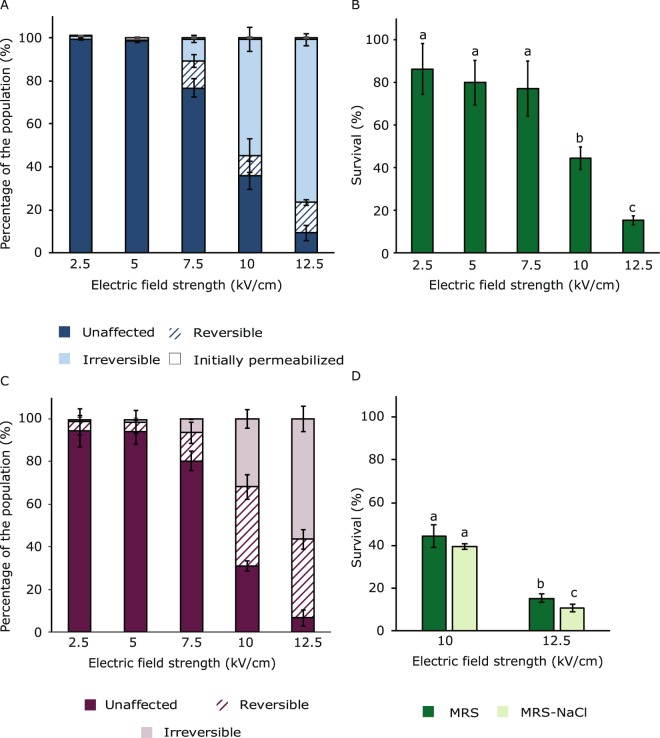
Membrane permeabilization and survival after PEF treatments at various electric field strengths. (**A**) membrane permeabilization assessed by PI addition before or after PEF treatment, (**B**) Survival after PEF treatment assessed by plate counting (adapted from Vaessen *et al*.^9^). (**C**) Membrane permeabilization assessed by combination staining with PI and SYTOX Green, (**D**) Survival assessed by plating on MRS and MRS-NaCl plates. For all PEF treatment two square wave pulses of 100 µs duration were applied. Error bars represent standard deviations of biologically independent samples (n ≥ 2) and different letters indicate significant differences (P < 0.05).

Membrane permeability of *L. plantarum* WCFS1 was evaluated for various electric field strengths by evaluating PI uptake before and after PEF treatment. Four different fractions could be distinguished; cells with an unaffected intact membrane after PEF, cells with a reversible permeabilized membrane for PI, cells with an irreversible permeabilized membrane and cells that were already permeable for PI before PEF (staining method 1 in Fig. 1). *L. plantarum* WCFS1 became permeable for PI after PEF treatment using an electric field strength of 7.5 kV/cm or higher when 2 pulses of 100 µs were applied (Fig. 2A). At 7.5 kV/cm approximately 10% of the population was reversible permeabilized and a similar percentage was irreversible permeabilized for PI. At higher electric field strengths, the irreversible permeabilized fraction of the population increased, while the reversible permeabilized fraction of the population remained approximately 10%. This increase in irreversibly permeabilized cells at higher electric field strengths is in line with the survival results based on plate counts (Fig. 2B). Also, it is in line with previous research on inactivation of microorganisms using PEF treatment, where a higher electric field strength led to an increased microbial inactivation^17,29,30^.

In order to apply reversible PEF treatment for loading of viable bacterial cells with small molecules, it is important that the reversible permeabilized cells survive the PEF treatment. However, we cannot draw a conclusion on the survival of the relatively small fraction of reversible permeabilized cells in Fig. 2A by comparing these results with survival results based on plate counts in Fig. 2B. The evaluation of plate counts on this percentage scale is limited due to the sensitivity of the plating method.

**Figure 3:**
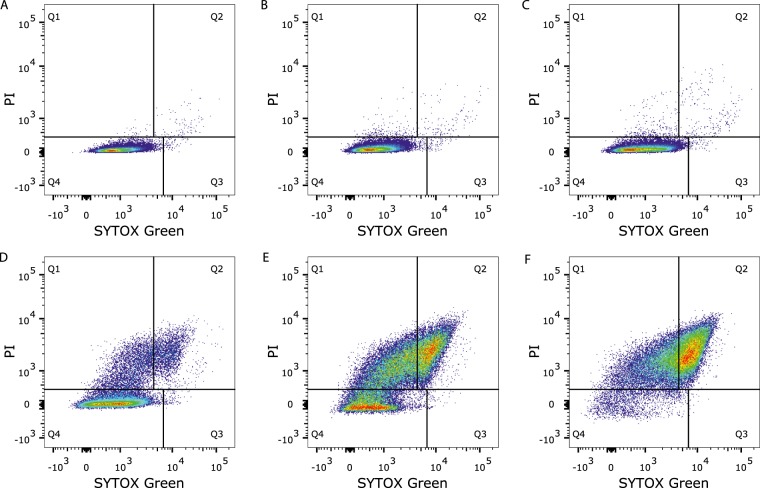
Fluorescence density plots obtained by flow cytometry of the bacterial population stained with PI and SYTOX Green without electroporation (**A**) and electroporated with two pulses of 100 µs at 2.5 (**B**), 5 (**C**), 7.5 (**D**), 10 (**E**) and 12.5 (**F**) kV/cm. Each dot represents a single bacterial cell. The colours represent a density scale from purple/blue (low) to red (high cell density). The quadrants represent the different fractions of the population: Q1: reversible permeabilized, Q2: irreversible permeabilized and Q4: unaffected intact membrane. These density plots are presented as an example from one of the experiments of which the results are presented in Fig. 2C.

The membrane permeability results of the second staining method, with the addition of PI before PEF and SYTOX Green after PEF treatment (Fig. 2C), resulted in very limited membrane permeability for electric field strengths up to 7.5 kV/cm, and was only slightly higher compared to the first method (Fig. 2A). Interestingly, at higher electric field strengths a larger fraction of reversible permeabilized cells was found with the double staining method. In this method, the different fractions were obtained using flow cytometry as can be seen in more detail in Fig. 3. Figure 2C shows that instead of a reversible permeabilized fraction of approximately 10% quantified using method 1, method 2 showed that this part of the population increased to approximately 40% at 10 kV/cm. This raises the question which of the two methods gives a better representation of the reversibility of membrane permeabilization upon PEF treatment.

As an additional control, also staining with SYTOX Green before and after PEF has been performed for PEF treatments at 7.5 and 10 kV/cm. These experiments at 10 kV/cm resulted in a reversible permeabilized fraction which was slightly larger than with the addition of PI before and after PEF, but smaller compared to the combination staining method. At 7.5 kV/cm no clear differences between the methods were observed (Supplementary Material S1). It could be reasoned that using a combination staining method is less suitable, because the two stains could compete for the same binding sites on the DNA. However, Müller *et al*. described that no displacement between SYTOX Green and PI was observed in their experiments when these stains were used together, indicating that these stains probably have different binding sites in the DNA^31^. Our own additional experiment with a mixture of live and dead cells confirmed this finding (Supplementary Material S2). Also, the decreased effectiveness of nucleic acid stains when DNA is damaged^32^ might play a role in the observed effect, as PEF could damage DNA^33^.

Another method which is often used to detect sublethal damage of bacteria during PEF treatment is selectivity plating^16^. Although this method is based on outgrowth rather than membrane permeability, it might give an indication of what is more likely; a large or small reversible permeabilized part of the population? Therefore, control and PEF treated samples at 10 and 12.5 kV/cm were plated on both MRS and MRS-NaCl plates. For both PEF conditions only small differences, which were significant only at 12.5 kV/cm, were observed in survival between MRS and MRS-NaCl plates (Fig. 2D). These small differences are more in line with membrane permeability based on only PI staining (method 1) than with the combination staining with PI and SYTOX Green (method 2). Based on a comparison of the double staining method (Fig. 2C) with the survival results (Fig. 2B,D), it can be concluded that not all the cells that were reversible permeabilized according to this double staining method were able to form colonies.

### Membrane permeabilization and population properties

A big advantage of the membrane permeability assessment with the double staining method with PI and SYTOX Green is the possibility for more in depth analysis on the characteristics of the three different subpopulations. The three subpopulations; namely intact cells, reversible permeabilized cells and irreversible permeabilized cells, are categorised based on the PI and SYTOX Green fluorescence intensities, as shown in the density plots in Fig. 3. As an extra check, the subpopulations have also been determined based on the separate PI and SYTOX Green signals, which resulted in similar percentages of the different fractions for all conditions (data not shown). The membrane permeability results that were derived from these density plots are shown in Fig. 2C.

As can be seen in Fig. 3, the increase in PI and/or SYTOX Green fluorescence intensity is not at the same level for every cell, but covers a range of signal intensities. With increasing the electric field strength, the population shifted towards higher PI and SYTOX Green intensities, indicating a shift from unaffected cells (low PI, low SYTOX Green) to reversible permeabilization (high PI, low SYTOX Green), and to irreversible permeabilization (high PI, high SYTOX Green).

**Figure 4:**
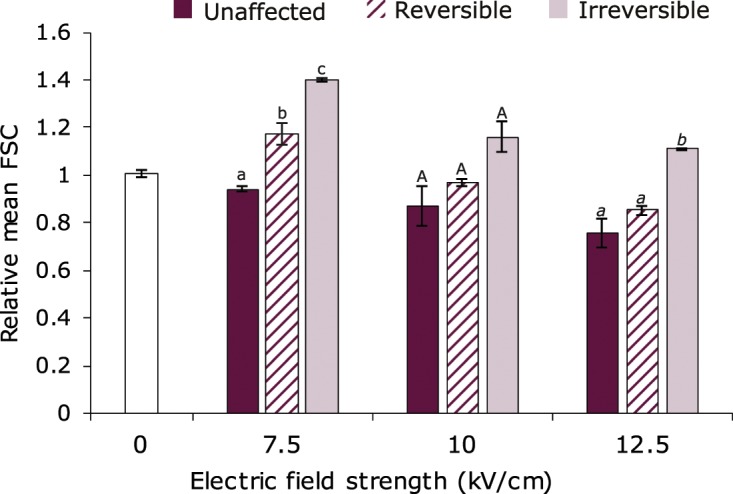
Relative mean forward scatter (FSC) signals for three different fractions of the population; unaffected intact cells, reversible permeabilized cells and irreversible permeabilized cells. The different groups are determined based on PI and SYTOX Green staining and relative mean FSC values are determined by dividing the mean FSC signals of specific populations by the mean FSC value of the entire cell population. The data point presented at 0 kV/cm is a control; namely the mean FSC of the control culture divided over the mean FSC of the PEF treated cultures. Error bars represent standard deviations of biologically independent duplicates and different letters indicate significant differences (P < 0.05) between the three fractions at one specific electric field strength.

Subsequently, the forward scatter (FSC) values, which are determined from the light scatter of a bacterial cell measured in the path of the laser, were assessed for the three different subpopulations. The FSC value can give an indication of the bacterial cell size, where lower FSC values correspond to smaller sized cells^34,35^. However, this relation between FSC and cell size does not always hold, since cell morphology might also affect FSC values. Interestingly, the mean and median FSC values differed between the three subpopulations. The intact cells that did not take up any stain after PEF had lower mean FSC values than the reversible permeabilized cells, which again had lower FSC values than the irreversible permeabilized cells (Fig. 4). This trend was observed at 7.5, 10 and 12.5 kV/cm, though these differences in FSC value were not in all cases significant. The data for the median FSC showed the same trends (data not shown). The relative mean FSC was calculated by dividing the specific FSC value of the subpopulation of interest by the FSC value of the entire population (unaffected + reversible + irreversible). As a control, the mean and median FSC values of the whole electroporated cell population were compared to those of a cell population without electroporation. The mean and median FSC values for both populations were similar, thus the electroporation treatment itself did not affect the FSC values. The FSC results showed larger mean and median FSC-values for the irreversible fraction, suggesting that the larger cells in the population are relatively more affected by the PEF treatment compared to the smaller cells. Wouters *et al*. observed a similar relation between FSC and PI uptake after electroporation in *Lactobacillus plantarum* LA10–11, though they did not take the reversible fraction into account, as PI was only added after electroporation^12^. Interestingly, the mean FSC value of the reversible permeabilized fraction was for all PEF conditions between the values for the irreversible and unaffected cell fractions. The underlying mechanism for this observation remains to be determined, though this observation triggered us to speculate about it. These results suggest that reversibility of permeabilization is due to a balance between the intensity of the PEF treatment and the bacterial cell size of the individual cells. It is probably not related to a specific fraction of the cells that more easily recovers their membrane integrity. In other words; the cells that were reversibly permeabilized after PEF treatment at 7.5 kV/cm were very likely to be irreversibly permeabilized at higher electric field strengths, and cells that were unaffected at 7.5 kV/cm might be reversible permeabilized at higher electric field strengths.

The FSC results in this study are in line with previous research on the relation between the efficacy of a PEF treatment and cell size, where in general it was observed that the larger the cell the more easy it is permeabilized upon a specific PEF treatment^36,37^. However, it should be noted that this relation between cell size and efficacy of PEF does not always hold when comparing different bacterial strains to each other^38^. Other factors such as pre-culture conditions, Gram-positive or Gram-negative or cell morphology can play a role as well.

### Effect of pulse parameters on reversibility of membrane permeabilization

**Figure 5:**
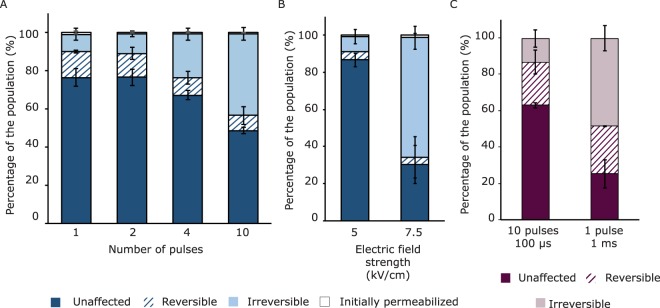
Membrane permeabilization upon PEF treatment assessed by PI staining before and after PEF (**A,B**) and by double staining with PI and SYTOX Green (**C**). (**A**) PEF treatments at 7.5 kV/cm with 100 µs pulse duration and varying number of pulses. (**B**) PEF treatment at two electric field strengths with one pulse of 1 ms duration. (**C**) PEF treatments at 7.5 kV/cm. Error bars represent standard deviations of biologically independent replicates (n ≥ 2).

In addition to electric field strength, other pulse parameters could influence reversibility of membrane permeabilization as well. Therefore, we studied the effect of the number of pulses and pulse duration on reversibility of membrane permeabilization. One, two, four and ten pulses of 100 µs at 7.5 kV/cm were applied (Fig. 5A), as well as one pulse of 1 ms at 5 and 7.5 kV/cm (Fig. 5B). Similar to the results presented in Fig. 2, the reversible permeabilized fraction remained small (maximum 10% of the population) for all these PEF conditions when the membrane permeability was assessed with the addition of PI before and after PEF treatment (Fig. 5A,B), and increase of the intensity of the treatment led only to an increase in the irreversible fraction. Other studies did find a larger reversible permeabilized fraction of the population reaching up to 80% in microalgae and mammalian cells^21,39^. Also for another Gram-positive bacterium, *Listeria monocytogenes*, reversible permeabilized fractions for PI reached up to 40% of the population^11^. However, it is noted that comparison with similar studies is not that obvious, since small differences in experimental set-up may lead to completely different outcomes, i.e. microbial and culture characteristics, PEF treatment medium, specific PEF conditions.

PEF treatments at 7.5 kV/cm with 10 pulses of 100 µs (Fig. 5A) and with one pulse of 1 ms (Fig. 5B) were compared having the same total pulse duration and similar energy input, but different number of pulses. Interestingly, one pulse of 1 ms resulted in more irreversible permeabilization compared to 10 pulses of 100 µs. One longer pulse is apparently more effective for irreversible membrane permeabilization than 10 shorter pulses at the same electric field strength, though the reversible permeabilized fractions for both conditions remained comparable at approximately 4–8% (Fig. 5A,B). This effect of more irreversible permeabilization using pulses of 1 ms compared to shorter pulses of 15 or 100 µs at the same energy input is in line with recent observations for inactivation of bacteria and yeast in fruit juices^40^. When comparing the same PEF conditions with the double staining method (Fig. 5C), a larger reversible permeabilized fraction was observed for this second method, similar to our earlier observations presented in Fig. 2. However, the difference between 10 pulses of 100 µs and 1 pulse of 1 ms remained similar with this method, showing that one pulse of 1 ms was more effective in terms of irreversible membrane permeabilization.

**Figure 6:**
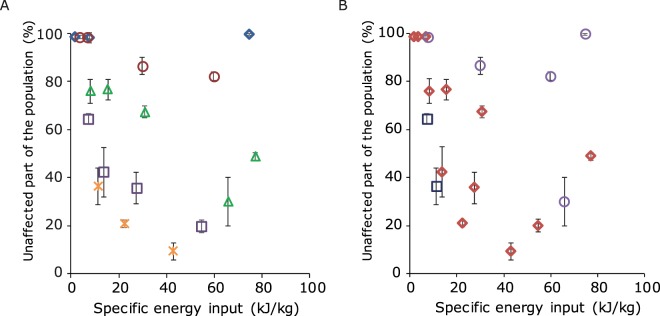
Percentage of the *L. plantarum* WCFS1 culture with an unaffected intact cell membrane after PEF treatment as assessed by PI staining for several PEF conditions. Both panels show the same experimental data presented in different ways. In figure A the symbols represent the different electric field strengths ( 2.5 kV/cm,  5 kV/cm,  7.5 kV/cm,  10 kV/cm,  12.5 kV/cm) and in figure B the different symbols represent the pulse duration of a single pulse ( 50 µs,  100 µs,  1 ms). Error bars represent standard deviations of biologically independent replicates (n ≥ 2).

The different settings of the PEF parameters, namely electric field strength, pulse duration and number of pulses applied in this study led to different energy inputs. The energy input for each PEF condition was calculated according to Eq. 1 and compared to the percentages of the population that remained unaffected (Fig. 6). Increasing energy input did not necessarily decrease the percentage of the population with an intact membrane. Also, the electric field strength played a role, as at the same specific energy input a higher electric field strength led to a lower number of cells with an intact cell membrane (Fig. 6A). These results confirm what has been found in studies on inactivation of bacteria and yeasts, where a higher electric field strength results in more microbial inactivation at a similar energy input^41,42^. Whereas a clear relation was observed between electric field strength and membrane permeability, this was not the case for pulse duration and membrane permeability (Fig. 6B). Here, a large variation in the percentage of the population with intact membrane was found for example for pulses of 100 µs with similar energy input. This variation shows that pulse duration is not the main PEF parameter affecting membrane permeability and confirms that changing the electric field strength has more influence.

Energy input can also be related to temperature increase (Eq. 2). Temperature increase during electroporation could potentially also contribute to cell inactivation and/or membrane permeabilization. The theoretical temperature increase at 100 kJ/kg energy input would be 25 °C, resulting in a theoretical temperature of approximately 45 °C. To assess whether the temperature increase alone would result in PI uptake, cultures of *L. plantarum* WCFS1 stained with PI were incubated in a water bath at 45 °C for 1 and 5 minutes. Also after 5 minutes still more than 98% of the cell population remained impermeable for PI (data not shown) and therefore the temperature increase alone is not considered to influence the membrane permeability results. However, an additive effect of the temperature increase to the PEF treatment, especially at higher energy inputs, cannot be entirely excluded based on the synergistic effect often described in PEF studies for inactivation purposes^43,44^.

Based on the membrane permeabilization results at different field strengths presented in Fig. 6A, there seems to be a critical electric field strength around 5 kV/cm that is required to permeabilize the membrane of *Lactobacillus plantarum* WCFS1. Based on previous research, a certain transmembrane potential difference is required for pore formation in the cellular membrane^45,46^. The transmembrane potential difference that corresponds to the observed critical field strength can be estimated from these field strengths in combination with the cell size and shape according to Eq. 3. In this equation, Δ*φ* is the transmembrane potential difference (V), *f*(*A*) a dimensionless shape factor for non-spherical cells, *A*_*F*_ the distance from the center of the cell to the cell membrane in the direction of the electric field (m) and *E* the electric field strength (V/m)^47,48^.

3 $$\Delta \phi =-f(A)\cdot {A}_{F}\cdot E$$

The shape factor can be approximated from the cell dimensions as described in Eq. 4, in which *l* is the length of the bacterial cell (m) and *d* the diameter (m)^49^.

4 $$f(A)=l/(l-0.33\cdot d)$$

Based on the cell size estimations for *L. plantarum* WCFS1 from microscopy pictures (being 1.9 µm × 0.8 µm) and when assuming the cell orientation to be in parallel with the direction of the electric field, a critical electric field strength of 5 kV/cm corresponds to a transmembrane potential difference of 552 mV. This transmembrane potential difference required for pore formation in the cell membrane has been determined before for several cell types and was found to be 240 mV for Chinese hamster ovary cells^5^, 150–500 mV for bilayer lipid vesicles^45^ and between 260–1300 mV for different bacteria depending on the pre-culture conditions and bacterial strain^49^. Additionally, a more recent microfluidic study found a critical electric field strength for SYTOX Green uptake around 5 kV/cm for the Gram positive bacterium *Corynebacterium glutamicum*^22^. Overall, the determination of the critical electric field strength and the transmembrane potential calculated from this field strength remain a rough approximation, since it depends on several assumptions, i.e. the cell orientation in the electric field and the method of evaluating PEF efficacy (e.g. fluorescent stains or plate counts). These assumptions, the cell types and pre-culture conditions differ between the studies and therefore should be considered when conclusions about a critical electric field strength or transmembrane potential difference to induce pore formation are made.

## Conclusion

Reversibility of membrane permeabilization upon PEF treatment was assessed with two different staining methods. One method was based on the use of a single stain, employing two parallel PEF treatments and the other one was based on a double staining approach using one PEF treatment. These two methods resulted in similar trends regarding the effect of the different PEF parameters on membrane permeabilization. Remarkably, clear differences between these two methods were found in the quantified fraction considered as reversible permeabilized. These differences indicate that the choice of fluorescent marker(s) can influence the conclusions drawn on the effect of a PEF treatment, e.g. the effect of PEF on enhancing molecule transport into the cells, advocating the use of different staining methods in parallel. Still, fluorescent staining techniques remain important for understanding the effect of specific PEF treatments on cells. The double staining method allowed us to also compare the forward scatter values of the different subpopulations, which provided an indication of differences in cell size and effect of permeabilization. Regarding the different PEF conditions evaluated in this study a critical electric field strength for membrane permeabilization is observed around 5 kV/cm for *L*. plantarum WCSF1. In addition, increase of the electric field strength above this critical value resulted in more membrane permeabilization when compared to other PEF conditions with a similar energy input.

## Supplementary information

Supplementary material

## Data Availability

The datasets generated during the current study are available from the corresponding author on reasonable request.
