# Supplementary material for: Reversibility of membrane permeabilization upon pulsed electric field treatment in Lactobacillus plantarum WCFS1
Source: Sci Rep. 2019 Dec 27;9:19990. doi: 10.1038/s41598-019-56299-w (PMC6934533; doi:10.1038/s41598-019-56299-w)
Supplement: Supplementary file 1 — Supplementary material [file 41598_2019_56299_MOESM1_ESM.docx]

**Supplementary material**

# Reversibility of membrane permeabilization upon pulsed electric field treatment in *Lactobacillus plantarum* WCFS1

E.M.J. Vaessen^1,2^, R.A.H. Timmermans^3^, M.H. Tempelaars^2^, M.A.I. Schutyser^1^, H.M.W. den Besten^2*^

^1^ Food Process Engineering, Wageningen University and Research, P.O. Box 17, 6700 AA Wageningen, The Netherlands

^2^ Food Microbiology, Wageningen University and Research, P.O. Box 17, 6700 AA Wageningen, The Netherlands

^3^ Wageningen Food and Biobased Research, Wageningen University and Research, P.O. Box 17, 6700 AA Wageningen, The Netherlands

*Corresponding author: [heidy.denbesten@wur.nl](mailto:heidy.denbesten@wur.nl)

1. **Third staining method: SYTOX Green before and after PEF**

As an additional control, some experiments have been performed with the addition of SYTOX Green before and after PEF treatment. The principle of this method is the same as the method with the addition of PI before and after PEF (described in the materials and methods section). The results presented in Fig. S1 were obtained from two biologically independent samples, for both replicates all three the staining methods have been performed and analysed by flow cytometry.

*Figure S1 Membrane permeabilization of L. plantarum WCFS1 after PEF treatment at 7.5 and 10 kV/cm assessed by three different staining methods, all analysed by flow cytometry.*

As can be seen in Fig. S1, the membrane permeability results at 7.5 kV/cm did not significantly differ between the three staining methods. However, at 10 kV/cm the addition of PI before and SYTOX green after PEF resulted in the largest reversible permeabilized fraction compared to the other two staining methods. Staining with SYTOX Green before and after PEF resulted in a slightly larger reversible permeabilized fraction compared to staining with PI before and after PEF, though not as large as with the double staining method.

1. **PI and SYTOX Green displacement?**

An initial staining experiment with a mixture of live and dead cells was performed to check the effect of the combination staining method (dead cells were heat treated at 70°C for 8 minutes). In these experiments we found that all dead cells clearly increased in both PI and SYTOX green signals as can be seen in Fig. S2, indicating that no (complete) displacement between these two stains took place.


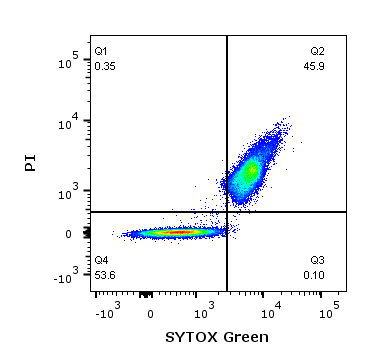


*Figure S2 Fluorescence density plot obtained by flow cytometry of a mixture of live and heat-killed L. plantarum WCFS1 cells stained with PI and SYTOX Green. Each dot represents a single bacterial cell. The colours represent a density scale from blue (low) to red (high cell density).*
